# Ketogenic diet in a patient with refractory status epilepticus due to *POLG* mutation

**DOI:** 10.1002/jmd2.12169

**Published:** 2020-10-01

**Authors:** Miriam Koessler, Edda Haberlandt, Daniela Karall, Matthias Baumann, Alexander Höller, Sabine Scholl‐Bürgi

**Affiliations:** ^1^ Department of Pediatrics I, Neuropediatrics Medical University of Innsbruck Innsbruck Austria; ^2^ Department of Pediatrics Hospital Dornbirn Dornbirn Austria; ^3^ Department of Pediatrics I Inherited Metabolic Disorders, Medical University of Innsbruck Innsbruck Austria

**Keywords:** ketogenic diet, *POLG* mutation, refractory status epilepticus

## Abstract

We present a 16‐year‐old female patient with *POLG* syndrome, treated with ketogenic diet after she presented with refractory status epilepticus. Initially, benefit of the ketogenic diet could be seen, but the outcome was fatal, with death 3 months after presenting symptoms. Additionally, we give a literature review of the utility of ketogenic diet in patients with *POLG* disease.

**Abbreviations**
cMRI: cerebral magnetic resonance imaging
EEG: electroencephalography
Lab: laboratory
LEV: levetiracetam
LZP: lorazepam
MDZ: midazolam
Mg: magnesium
MPL: methylprednisolon
PB: phenobarbital
PHT: phenytoin
POLG: polymerase gamma
TPM: topiramate
VPA: valproic acid

## 1 INTRODUCTION

Polymerase gamma (*POLG, 174763)* mutation leads to mitochondrial DNA depletion syndrome, resulting in epilepsy, movement disorders, cognitive impairment and liver dysfunction.^1^ Usually, clinical symptoms commence in early childhood, but later manifestation is also possible. Treatment options are limited and consist in symptomatic treatment. Beneficial effects of ketogenic diet were previously reported in six patients with *POLG* mutation.^2^, ^3^, ^4^, ^5^, ^6^ Ketogenic diet is based on high‐fat and low carbohydrate intake. This induces a shift in cellular energy supply from glucose to ketones. In inborn disorders of metabolism, it is used in two different ways, firstly, targeting mainly the underlying metabolic disorder or secondly, targeting mainly the clinical symptoms (eg, seizures/epilepsy).^7^ However, long‐term outcome is poor. Here we present a case of POLG syndrome treated with anticonvulsants and ketogenic diet with initial good response, but nevertheless poor long‐term outcome.

## 2 CASE REPORT

**FIGURE 1:**
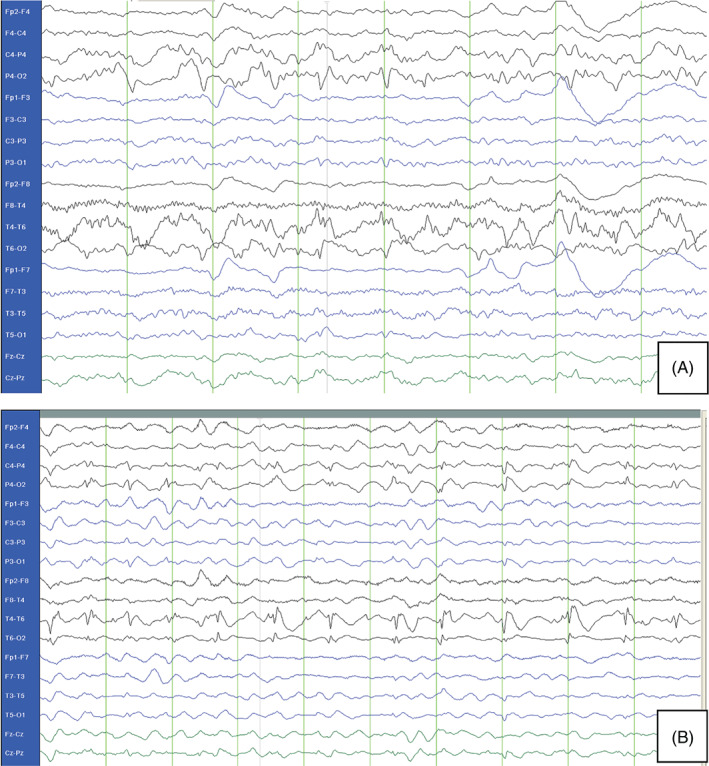
A, Initial EEG with focal slowing at admission. B, Control EEG 21 days after admission: diffuse generalized slowing, with continuous spike–wave activity temporo‐occipital and polyspikes‐compatible or pathognomic for *POLG* mutation

A previously healthy 16‐year‐old female juvenile presented with migraine type headache. Within the outpatient visit, a secondary generalized seizure occurred. Cerebral MRI (cMRI) showed decreased diffusion on right occipital lobe; electroencephalography (EEG) presented focal slowing (Figure 1). On the same day, the patient fell into an intractable focal status epilepticus, requiring intensive care treatment. Therapy with lorazepam, levetiracetam, phenytoin, cortisone, valproate and lacosamid could not interrupt focal status epilepticus.

Diagnostic work‐up included lumbar puncture, which showed an isolated blood‐cerebrospinal fluid barrier dysfunction (leukocytes 3/μL [0‐4/μL], protein 659 mg/L [150‐450 mg/L] and albumin quotient 9.2 [<8]). In view of the clinical neurological symptoms, such as ascending tetraparesis, the suspicion of Guillain‐Barre‐syndrome raised and immunoglobulins IV were administered over 4 days without improvement.

Six days after admission and because of typical pattern of epilepsia, presenting as epilepsia partialis continua, POLG molecular genetic testing was performed (Dr. J.A. Mayr, Salzburg) and revealed homozygous *POLG* mutation. A missense mutation in the encoding region and the adjoining introns leading to c.1399G>A (p.Ala467Thr) exchange was identified. Parents, both heterozygous carriers of this mutation, are healthy.

This mutation has been previously described in patients with Alpers disease^8^, ^9^ (**#** 203700). Though our patient received valproic acid (for 2 days) before diagnosis, no reparable liver dysfunction was noted.

Immediately after diagnosis (day 9 after admission), in addition to antiseizure drugs, classical ketogenic diet (4:1) (KD) was started. Full ketosis (beta‐hydroxybutyrate in plasma >2 mmoL/L) could be achieved after 5 days. Laboratory tests showed high levels of liver transaminases in blood with a normalization within 4 days (maximum aspartate transaminase 704 U/L, alanine transaminase 782 U/L, gamma glutamyltransferase 1700 U/L), blood glucose levels and pH were within normal range (glucose 74‐110 mg/dL, pH 7.40‐7.45). Repeated abdominal ultrasounds were normal.

**FIGURE 2:**
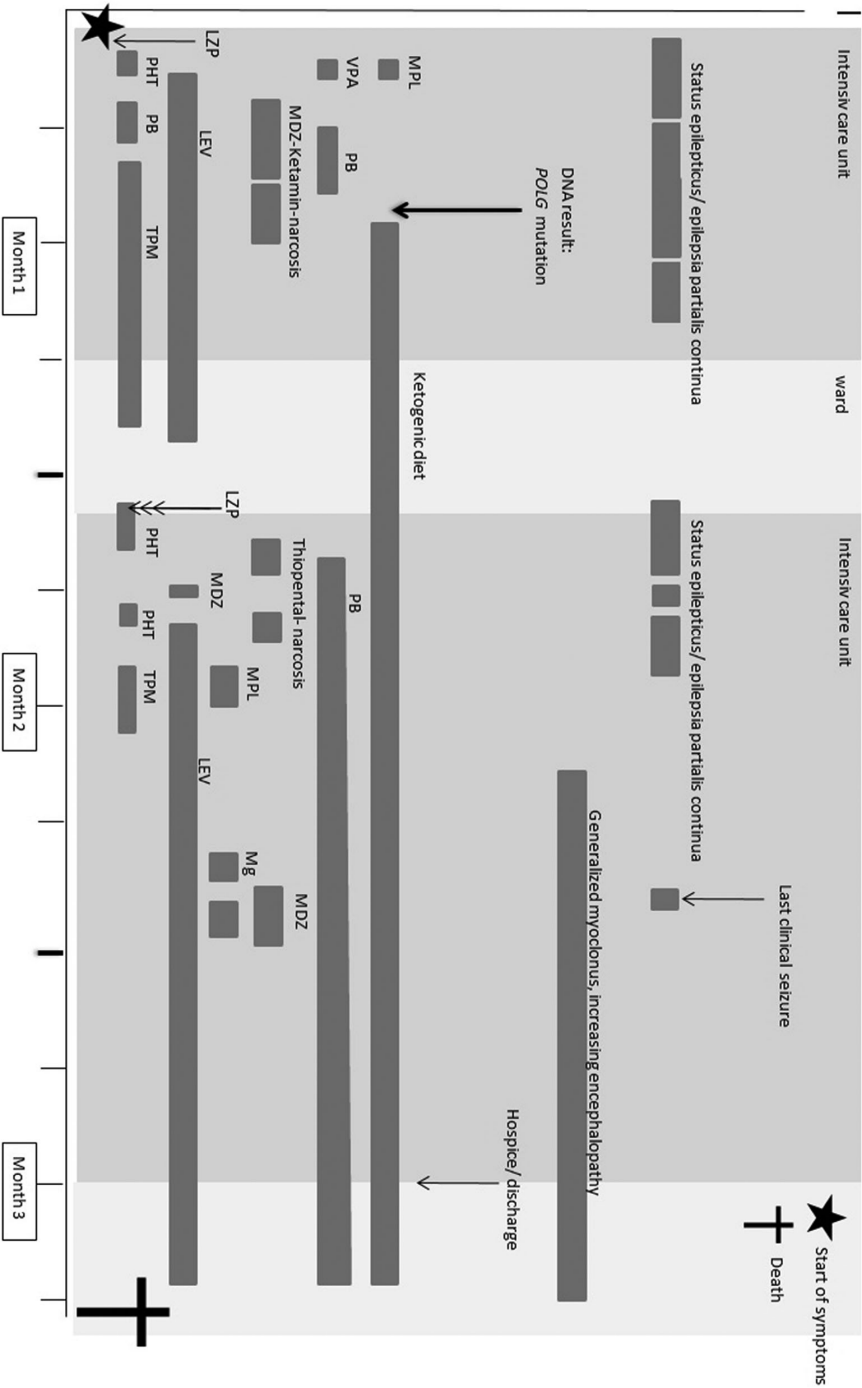
Timetable of our case report, with symptoms and treatments. LEV, levetiracetam; LZP, lorazepam; MDZ, midazolam; Mg, magnesium; MPL, methylprednisolon; PB, phenobarbital; PHT, phenytoin; TPM, topiramate; VPA, valproic acid. Maximum drug doses: levetiracetam, 60 mg/kg/d; lorazepam, 0.05 to 0.1 mg/kg per dose; ketamin, 3.5 mg/kg/h; midazolam, 0.08 to 0.2 mg/kg per dose, 0.3 mg/kg/h; phenobarbital, 10 mg/kg per dose, up to 50 mg/kg/d; phenytoin, 12 mg/kg/d; thiopental, 5 mg/kg/h; valproic acid, 40 mg/kg/d

First control cMRI performed 2 days after onset of symptoms showed alternating areas of decreased diffusion (altogether eight MRIs were done). EEG abnormalities were persistent with focal slowing and superimposed epileptic spikes as typical for patients with *POLG* mutations (see Figure 1). Only treatment with midazolam and ketamine narcosis in combination with phenobarbital could stop the status epilepticus after 25 hours, though reduction of narcosis was only feasible after initiation of ketogenic diet (on fifth day of narcosis, see Figure 2). Her condition stabilized and she could be transferred to regular ward.

Again 3 weeks later, she developed focal status epilepticus, despite ongoing therapy with levetiracetam, topiramate and phenytoin as well as ketogenic diet (4:1). Under treatment with thiopental narcosis for 5 days, seizures improved only short‐term. When sedation was tempered, she showed focal seizures despite intensive antiepileptic therapy (levetiracetam, phenobarbital, midazolam, phenytoin, topiramate and cortisone), magnesium infusion, ketogenic diet and riboflavin, coenzyme Q10 and thiamine.

Her condition worsened as she was severely encephalopathic and hardly able to communicate. She developed generalized myoclonus that did not correspond with an electroencephalographic change, and she had severe muscle pain.

Ten weeks after admission, she was transferred to a hospice, and finally going home with mobile home‐nursing. The patient died 3 months after presenting with initial symptoms (Figure 2) from apnea.

## 3 LITERATURE REVIEW

Only few reports exist on patients with *POLG* mutation and ketogenic diet (see Table 1).

**TABLE 1:** Review of patients with *POLG* mutation in literature, treated with ketogenic diet

	Joshi et al^2^	Cardenas and Amato^3^	Spiegler et al^4^		Martikainen et al^5^	Khan et al^6^	Koessler et al 2020
Gender	Female	Female	Male	Female	Female	Male	Female
*POLG* mutation	c.2243G>C; c.2480+1g>A	c.911T>G, c.1174C>g, pR1081dup	c.911T>G, c.3434insGAGG	c.844T>G, c.1399G>A	c.2243G>C	c.1399G>A; c.3562T>C	c.1399G>A
Age at initial presentation	31 mo	14 mo	15 mo (DD) 27 mo (SE)	18 mo (DD) 43 mo (SE)	22 y	—	16 y
Age at diagnosis	55 mo	14 mo	33 mo	45 mo	26 y	9 mo	16 y
Age at death	66 mo	19 mo	35 mo	46 mo	—	14 mo	16 y
Start of KD	55 mo	14 mo	33 mo, stopped 2 weeks later	NA	LGIT	13 mo	After diagnosis
Co‐medication	LEV, ESM, Nitrazepam	Multidrug	NA (VPA over a short period)	TPM, LCM (VPA over a short period)	PHT, OXC, LEV		LEV, PHT, LCM
Clinical improvement	Yes	Yes	No	Yes	Yes	Yes	Initially

Abbreviations: CLB, Clobazam; CBZ, Carbamazepin; DD, developmental delay; ESM, Ethosuximid; KD, Ketogenic diet; LCM, Lacosamide; LEV, Levetiracetam; LGIT, low glycaemic index therapy; NA, not available; OXC, oxcarbazepine; PB, phenobarbital; PHT, phenytoin; SE, status epilepticus; TPM, topiramate; VPA, valproic acid.

In 2009, Joshi et al described a 4.5‐year‐old girl with Alpers‐Huttenlocher syndrome and a heterozygous mutation in *POLG1* gene (c.2243G>C; c.2480+1g>A).^2^ The girl presented with epilepsia partialis continua, after initiation of ketogenic diet (4:1) she remained seizure‐free for 7 months. Triggered by an intercurrent infection under diet, she showed a subclinical status epilepticus, which could be successfully treated with midazolam infusion. After restart of the diet neither seizure control nor her baseline clinical state could be sustained anymore. At the age of 5.5 years, and 11 months after diagnosis, she died due to respiratory failure.^2^

One year later (2010), Cardenas and Amato described a 14‐month‐old girl with compound heterozygous mutation in *POLG1* gene (c.911T>G, c.1174C>g, pR1081dup) presenting with epilepsia partialis continua evolving into generalized status epilepticus. Treatment with a multidrug therapy, including ketogenic diet terminated her seizures, but she was severely encephalopathic. Several weeks after discharge seizures returned and she died at the age of 19 months, 5 months after diagnosis.^3^

In 2011, Spiegler et al reported two patients diagnosed with Alpers‐Huttenlocher syndrome due to *POLG1* mutation (patient 1 c.911T>G, c.3434insGAGG; patient 2 c.844T>G, c.1399G>A) at ages 33 and 45 months. Both were treated with ketogenic diet. The first patient did not respond and treatment was stopped 2 weeks after initiation, the boy died 3 months after diagnosis, at 35 months of age. The second patient became more alert and seizure activity ceased for a few weeks, but developed epilepsia partialis continua under treatment with antiepileptic drugs and KD, therapy was continued until death at 46 months.^4^

A 26‐year‐old woman with homozygous mutation in *POLG1* gene (c.2243G>C) and non‐convulsive status epilepticus was presented by Martikainen et al.^5^ Under low glycemic index treatment (LGIT), a variant of ketogenic diet, in addition to phenytoin, oxcarbazepine and levetiracetam, her symptoms resolved. Phenytoin and oxcarbazepine were gradually discontinued and she had no further seizures under levetiracetam monotherapy and LGIT.^5^

Another group described a boy diagnosed with Alpers syndrome and heterozygous *POLG1* mutation (c.1399G>A; c.3562T>C) at the age of 9 months.^6^ After starting ketogenic diet at 13 months of age, seizures substantially decreased without clinical improvement. One month later and 5 months after diagnosis, he died due to congestive heart failure and respiratory difficulties.^6^

## 4 DISCUSSION

### 4.1 Patients

In our literature research, we found six patients with *POLG* mutation and intractable seizures receiving ketogenic diet^2^, ^3^, ^4^, ^5^ (see Table 1). In five of these six patients, the diet led to substantial reduction of seizure activity, whereas general condition often did not improve.^2^, ^3^, ^4^, ^5^, ^6^ Four of these five died shortly after initiation of therapy^2^, ^3^, ^4^, ^6^ (see Table 1). One reported patient did not respond, hence therapy was stopped after 2 weeks^4^ (see Table 1).

In our patient, ketogenic diet only showed initial improvement of symptoms, with an improvement of EEG, finally leading to a reduction of antiseizure therapy and a stabilization of her general condition over a short time. However, she showed an outstanding rapid deterioration leading to death 3 months after initial presentation.

### 4.2 Ketogenic diet

Ketogenic diet is used in inborn disorders of metabolism^10^, ^11^ and is the treatment of choice in some of them; for example, glucose transporter type 1 deficiency syndrome.^12^, ^13^, ^14^ Further, ketogenic diet was found to be effective for therapy of intractable seizures.^11^, ^15^, ^16^ However, the precise mechanism of action of the diet is not fully understood. As some inborn disorders of metabolism deteriorate with catabolism, ketosis should be reached and maintained without catabolism.

According to data,^1^ most patients with *POLG* mutation presented with first symptoms in their early childhood. Our patient showed a juvenile and rapid progressive form of *POLG* mutation. The group of Martikainen presented a woman at the age of 26 years.^5^ Beside the adult patient, all patients died within 1 year after diagnosis. Thus, one might conclude that despite an initial improvement with ketogenic diet outcome is poor once symptoms have started.

## AUTHOR CONTRIBUTIONS

Miriam Koessler designed and implemented the article, and wrote the manuscript. Edda Haberlandt, Daniela Karall, Matthias Baumann have made substantial contribution to conception, design and interpretation of data. Sabine Scholl‐Bürgi has made substantial contributions to conception and design, acquisition and interpretation of data. Alexander Höller A contributed to the conception. All authors read, revised the manuscript critically and approved the version of the manuscript.
